# Effect modification by gender and smoking status on the association between obesity and atopic sensitization in Chinese adults: a hospital-based case–control study

**DOI:** 10.1186/1471-2458-14-1105

**Published:** 2014-10-26

**Authors:** Xiao Luo, Yupeng Wang, Zhiqiang Wang, Xiao-hua Zhou, Jing Zhao, Jianing Suo, Xiaohui Dong, Meina Liu

**Affiliations:** Public Health College, Harbin Medical University, 157 Baojian Road, Harbin City, Heilongjiang Province Postcode 150081 P.R. China; School of Medicine, the University of Queensland, Room 817, Health Sciences Building, Royal Brisbane & Women’s Hospital, Herston, QLD 4029 Australia; Department of Biostatistics, School of Public Health, University of Washington, Seattle, WA 98198 USA

**Keywords:** Obesity, Body mass index, Atopy, Adult

## Abstract

**Background:**

There is an ongoing debate on the potential association between obesity and atopy. However, no previous studies have investigated whether this relationship depends on sex and smoking status in Chinese adults.

**Methods:**

In this hospital-based, case–control study, we recruited 1150 atopic cases aged 18 years or older and 1245 healthy control participants during April 2009 and December 2012 in Harbin, China. We conducted structured questionnaire interviews, anthropometry measurements and serum allergen-specific immunoglobulin E (IgE) testing. Univariate and multivariate logistic regression models were used to explore the relationship between obesity and atopy risk stratified by sex and smoking status.

**Results:**

There was an association between obesity and an increased risk of atopic sensitization after adjusting for age, educational, family history, smoking and alcohol consumption (OR: 2.61, 3.25; 95% CI: 1.57-4.33,1.91-5.56 in males and females, respectively). The association between BMI and allergic sensitization depended on smoking status. In both genders, the association of obesity with atopic sensitization risk was stronger in non-smokers than in current smokers. In males, ORs of atopic sensitization for obesity were 3.15 (95% CI, 1.46-6.68) for non-smokers and 2.22 (95% CI, 1.10-4.48) for current smokers. The corresponding ORs in females were 3.51 (95% CI, 1.98-6.24) and 2.22 (95% CI, 0.46-10.68) for non-smokers and current smokers, respectively. After excluding those subjects who with pre-existing allergic conditions, the same relationship still remained.

**Conclusions:**

Obesity is positively and significantly associated with the risk of atopy in both men and women as well in both smokers and non-smokers in China. In addition, the relationship between obesity and atopic sensitization is stronger in non-smokers than in current smokers.

## Background

The prevalence of allergic diseases has increased in recent years [1–3], especially in low and middle income countries like China [4, 5]. Atopy refers to a hereditary predisposition of some individuals to develop immunoglobulin E antibodies (IgE) to common allergens [6], commonly preceding the development of allergic diseases [7] and usually defined as having detectable allergen specific IgE either by serum IgE tests, skin prick tests or other laboratory examinations. Considering the rapid rise of allergic disorders, understanding the associations of potential factors with atopic sensitization has important clinical and public health implications.

Obesity has been linked to the increase risk of atopic sensitization in some epidemiological studies [8–12], while other studies demonstrated that there is no association between obesity and atopic sensitization [13–17]. The reasons for these contradictory findings might be due to differences in obesity definitions, laboratory methods, populations and sample sizes. For instance, in a study of Chinese men and women, no association was found between body mass index (BMI) and allergic sensitization, whereas percent body fat (%BF) was found to be associated with a higher rate of allergic sensitization in men but no associations between %BF and any sensitization in women [10]. In another study, obesity was associated with a higher risk of atopy, and this association was largely driven by food sensitization [12]. Some studies have found association between obesity and allergic sensitization, while others have not. Gender might also play an important role in the association between obesity and atopic sensitization. From a biological view, sex hormone may contribute to the increased risk of atopy [18]. Moreover, females have higher amount of body fat than males [19], which might increase the risk of atopic sensitization in females, but epidemiological evidence is still inconsistent [9, 10]. Therefore, it is necessary to clarify the relationship between obesity and atopic sensitization stratified by gender.

Cigarette smoking may reduce atopic sensitization, and this association was confirmed in several cross-sectional and longitudinal studies, even though some bias cannot be ruled out such as healthy smoker bias and self-selection bias [20–27]. Consistent with previous studies, a 32-year well designed population-based cohort study supported the hypothesis that the suppressive effects on immune system of smoking protect against atopy [27]. Therefore, the negative relationship between smoking and the incidence of allergic sensitization could partly explained by an immunosuppressive effect. Considering that adipose tissue can also be involved in inflammatory processes [28], there might be a complex relation between body weight and smoking status, which could affect the incidence of allergic sensitization. On the one hand, due to the increased metabolic rate [29–31] or restricting caloric absorption [32], body weight in current smokers is generally less than that in never smokers. This may reduce the risk of obese individuals with increased atopic sensitization. On the other hand, ex-smokers often gain weight after smoking cessation [33–36], which may cause a higher risk of atopic sensitization. Therefore, the association between body weight and atopic sensitization may be modified by smoking status. However, as far as we know, no previous studies have examined the relationship between obesity and IgE-mediated allergen sensitization by smoking status in Chinese adults.

In this hospital-based case control study, we aimed to investigate the association between obesity and atopic sensitization among Chinese adults. We are particularly interested in exploring this association between obesity and atopy stratified by gender and smoking status. Understanding whether the association between obesity and atopy depends on gender and smoking status is important for developing tailored intervention strategies.

## Methods

### Study participants

This is a hospital-based, case–control study conducted at the Allergy Department of First Affiliated Hospital of Harbin Medical University in Harbin, China. Written informed consents were obtained from all subjects involving in this study. This study was approved by the Human Ethics Review Board, Harbin Medical University. (Reference number is HMUIRB20120019).

From April 2009 to December 2012, all subjects who visited the Department of Allergy in the First Affiliated Hospital of Harbin Medical University underwent the specific IgE testing regardless of their pre-existing conditions. Those aged 18 years or older were eligible for this study. Cases and controls were identified based on the results of the specific IgE testing. We identified cases including those who were newly diagnosed atopy based on positive specific IgE testing to at least 1 of the 13 most common local aeroallergens and food allergens. Those cases who had been previously diagnosed with allergic conditions were also confirmed positive in at least one of the IgE tests. Potential controls included healthy adults who visited the same department of the hospital for a health check-up during the same period. Final eligible controls were those who were negative in all specific IgE tests, and had not been previously diagnosed of any allergic diseases (included allergic eczema, allergic asthma, allergic rhinitis and food allergy). Among cases and controls, athletes, those currently undergoing professional anaerobic training, and pregnant or breastfeeding women were excluded because their obesity status could not be appropriately determined using body mass index. Subjects who did not take serum allergen-specific IgE testing, were not willing to complete the questionnaire interview or anthropometry measurements were also excluded from the study. In addition, we excluded those subjects who were ex-smokers due to small number of ex-smokers(men, 25 cases and 44 controls; women, 13 cases and 16 controls) for estimating the association between obesity and atopic sensitization. Finally, our analysis included the data from 2395 participants (1150 atopic subjects and 1245 non-atopic subjects).

### Questionnaire

A face-to-face interview was conducted by trained interviewers. One page questionnaire included personal demographic factors (sex, age, educational level), family history (parents, siblings, and children with related diseases including allergic asthma, allergic rhinitis, allergic eczema and food allergy), smoking status and alcohol consumption. Age was calculated as the difference between the year of birth and the year of interview. Educational level was categorized into three groups: illiterate/elementary school, junior/senior high school, and college, university or higher. Current smokers were defined on the basis of the World Health Organization criteria, as those who self-reported smoking every day for at least 6 months [37]. Regular alcohol drinkers were defined as drinking more than twice per week for at least one year.

### BMI calculation and classification

According to a standard protocol [38], we measured weight and height of all participants. Subjects were required to dress in normal indoor clothing and without shoes, weighed to the nearest 0.1 kg using a calibrated standard scale. Height was measured to the nearest 0.1 cm using a stadiometer (Detecto-Scales; Brooklyn, NY). The physician who conducted anthropometry did not know the purpose of this study. BMI was calculated as weight (kg)/[height (m)]^2^. All subjects were grouped into one of the following three groups, normal weight (BMI < 24), overweight (BMI ≥ 24 and BMI < 28) and obesity (BMI ≥ 28) according to the criteria established in 2003 by the Working Group on Obesity in China [39].

### Allergen-specific IgE testing and atopy

Serum samples were tested for allergen-specific IgE using the AllergyScreen system (Mediwiss Analytic GmbH, Germany). The choice of allergens was based on the epidemiology of atopic sensitization in China. The thirteen most common allergens in the northeast of China were assessed, including seven aeroallergens and six food allergens. Aeroallergens include *Dermatophagoides pteronyssinus*, common ragweed and mugwort, Hop, cat and dog fur, mould mixture (*Penicillium notatum, Cladosporium herbarum, Aspergillus fumigatus, Alternaria alternate*), tree pollen mixture (*Robur, Elm, London Plane, Willow, Cottonwood*) and German cockroach. Food allergens include egg white/egg yolk, fish, crab, shrimp, milk and beef. Atopy was defined as if the concentration of a least one allergen-specific IgE was 0.35 kU/L or greater.

### Statistical analysis

The baseline characteristics of cases and controls were examined. Age was categorized into three groups: 18–39,40-59, ≥60 years. The associations of categorical variables with atopy were estimated using chi-squared tests. To assess the associations of obesity with atopic sensitization and to control for the potential confounding factors, crude and adjusted odds ratio (OR) and their 95% confidence intervals (CI) were calculated using logistic regressions. Separate analyses were conducted for different genders and smoking status. A two-sided *p* value of less than 0.05 was taken to indicate statistical significance for all estimates. The effect modifications of gender and smoking status on a multiplicative scale were assessed by including interaction terms between BMI and gender or smoking status in logistic regression models. A *p* value for the interaction term of less than 0.10 was considered evidence of interaction. Stratified models were used to assess the associations between BMI and atopy where evidence for interaction was found. The *p* values for linear trends were calculated by entering the ordinal variable of BMI as a continuous term in the regression model. All analyses were performed using statistical SAS 9.1 (SAS Inc., Cary, NC, USA).

## Results

In the present study, a total of 2395 participants were recruited, including 361 men cases and 384 men controls, 789 women cases and 861 women controls. The numbers and percentage of demographic characteristics, smoking status of cases and controls by gender are shown in Table 1. Both men and women cases were more likely to have higher educational level than controls and to have higher proportion of obesity than controls. In addition, controls were more likely than cases to be older in men. However, age difference was not seen in women. With regard to family allergic disease history, smoking status and regular alcohol drinking status, the differences between atopic sensitization group and non-atopic sensitization group were not statistically significant.

**Table 1:** **Characteristics of atopic sensitization cases and hospital health controls in men and women**

	Men			Women		
	Case	Control	P value	Case	Control	P value
	N (%)	N (%)		N (%)	N (%)	
**N**	361	384		789	861	
**Age (years)**			0.0286			0.0778
18-39	212 (58.73)	189 (49.22)		435 (55.13)	433 (50.29)	
40-59	128 (35.46)	163 (42.45)		315 (39.92)	391 (45.41)	
≥60	21 (5.82)	32 (8.33)		39 (4.94)	37 (4.30)	
**Educational level**			0.0004			<0.0001
1st	88 (24.38)	60 (15.63)		176 (22.31)	105 (12.20)	
2nd	129 (35.73)	188 (48.96)		313 (39.67)	492 (57.14)	
3rd	144 (39.89)	136 (35.42)		300 (38.02)	264 (30.66)	
**Family allergic disease history**			0.6750			0.6302
No	304 (84.21)	319 (83.07)		628 (79.59)	677 (78.63)	
Yes	57 (15.79)	65 (16.93)		161 (20.41)	184 (21.37)	
**Smoking status**			0.6383			0.8020
No	212 (58.73)	232 (60.42)		739 (93.66)	809 (93.96)	
Current smoking	149 (41.27)	152 (39.58)		50 (6.34)	52 (6.04)	
**Regular alcohol drinkers**			0.6501			0.8944
No	216 (59.83)	236 (61.46)		748 (94.80)	815 (94.66)	
Yes	145 (40.17)	148 (38.54)		41 (5.20)	46 (5.34)	
**BMI**			0.0016			<0.0001
Normal	178 (49.31)	224 (58.33)		582 (73.76)	629 (73.05)	
Overweight	129 (35.73)	132 (34.38)		149 (18.88)	212 (24.62)	
Obesity	54 (14.96)	28 (7.29)		58 (7.35)	20 (2.32)	

Table 2 shows the associations between BMI classification and atopic sensitization in males and females. The strength of the association between obesity and atopic sensitization depended on sex with a significant sex-BMI interaction term (*p*_effect *modification*_ *=* 0.0414). Compared to men with normal BMI, obese men had a notably increased risk of atopic sensitization after adjusting for age, educational, family history, smoking and alcohol consumption (OR, 2.61; 95% CI, 1.57-4.33). This association is stronger in women (OR, 3.25; 95% CI, 1.91-5.56), after adjusting for the same set of confounding factors. In order to exclude allergic induced obesity, such as those with reduced physical activity or side reaction of therapy related to allergic diseases, we excluded subjects with previously diagnosed allergic diseases. The strength of association between BMI and atopic sensitization varied between men and women. Obesity was positively associated with atopic sensitization with OR = 2.76 (95% CI, 1.54-4.95) in men, 2.93 (95% CI, 1.59-5.40) in women. Interestingly, overweight women had a lower risk of atopic sensitization than women with normal BMI with OR = 0.76 (95% CI, 0.60-0.96). Even after adjusting for other confounding factors, this association still existed (OR, 0.76; 95% CI, 0.59-0.97). However, when we excluded those women with prior allergic diseases, this association was no longer statistically significant at the 5% level (OR, 0.76; 95% CI, 0.56-1.02).

**Table 2:** **ORs and 95% CIs for atopy in relation to BMI category stratified by sex in all subjects and those without allergic diseases**

	Men				Women				Effect
	Case	Control	cOR (95% CI) ^a^	aOR (95% CI) ^b^	Case	Control	cOR (95% CI) ^a^	aOR (95% CI) ^b^	modification
	N (%)	N (%)			N (%)	N (%)			P value ^c^
**All participants**									
Normal	178 (49.31)	224 (58.33)	1.00 (ref)	1.00 (ref)	582 (73.76)	629 (73.05)	1.00 (ref)	1.00 (ref)	
Overweight	129 (35.73)	132 (34.37)	1.23 (0.90-1.68)	1.29 (0.93-1.79)	149 (18.88)	212 (24.62)	0.76 (0.60-0.96)	0.76 (0.59-0.97)	0.0414
Obesity	54 (14.96)	28 (7.29)	2.43 (1.47-3.99)	2.61 (1.57-4.33)	58 (7.35)	20 (2.32)	3.13 (1.86-5.28)	3.25 (1.91-5.56)	
***P trend***				*p* = 0.0003				*p* = 0.0909	
**Without allergic diseases**									
Normal	97 (47.55)	224 (58.33)	1.00 (ref)	1.00 (ref)	334 (73.89)	629 (73.05)	1.00 (ref)	1.00 (ref)	
Overweight	76 (37.25)	132 (34.37)	1.33 (0.92-1.92)	1.41 (0.95-2.08)	89 (19.69)	212 (24.62)	0.79 (0.60-1.05)	0.76 (0.56-1.02)	0.0641
Obesity	31 (15.20)	28 (7.29)	2.56 (1.46-4.49)	2.76 (1.54-4.95)	29 (6.42)	20 (2.32)	2.73 (1.52-4.90)	2.93 (1.59-5.40)	
***P trend***				*p* = 0.0006				*p* = 0.3106	

^a^cOR, crude odds ratio; CI, confidence interval.

^b^aOR, adjusted odds ratio; CI, confidence interval.

^b^Adjusted for age, educational levels, family allergic diseases history, smoking status and alcohol consumption.

^c^P value for effect modification by sex on the association of BMI category and atopic sensitization after adjustment.

Considering the different smoking rate between males and females, we assessed the associations between BMI category and atopy risk stratified by smoking status for males and females separately. The association estimates between BMI category and atopic sensitization risk stratified by smoking status in each gender are shown in Table 3. In men, the associations between BMI category and atopic risk were statistically significant in non-smokers (*p*_trend_ *=* 0.0245) and current smokers (*p*_trend_ *=* 0.0042) while in women these trends were not statistically significant. Smoking status modified the effect of BMI category on atopy risk (*p*_*effect modification*_ *=* 0.0349 and 0.0260 for men and women, respectively). The OR of atopic sensitization for obesity in men was slightly lower in current smokers (2.22; 95% CI, 1.10-4.48) than that in never smokers (3.15; 95% CI, 1.49-6.68). Obesity was positively associated with atopy (OR, 3.51; 95% CI, 1.98-6.24) in never smoking women, but was not statistically significant in current smokers (OR, 2.22; 95% CI, 0.46-10.68). Nevertheless, the point estimates of OR indicated that obesity doubled the odds of allergic sensitization regardless of their gender and smoking status.

**Table 3:** **ORs and 95% CIs for atopy in relation to BMI stratified by sex and smoking status in all subjects**

		Never smokers			Current smokers				Effect
	Case	Control	cOR (95% CI) ^a^	aOR (95% CI) ^b^	Case	Control	cOR (95% CI) ^a^	aOR (95% CI) ^b^	Modification
	N (%)	N (%)			N (%)	N (%)			P value ^c^
***Men***									0.0349
Normal	102 (48.11)	118 (50.86)	1.00 (ref)	1.00 (ref)	76 (51.01)	106 (69.74)	1.00 (ref)	1.00 (ref)	
Overweight	82 (38.68)	102 (43.97)	0.93 (0.63-1.38)	1.02 (0.67-1.55)	47 (31.54)	30 (19.74)	2.19 (1.27-3.77)	2.15 (1.23-3.77)	
Obesity	28 (13.21)	12 (5.17)	2.70 (1.31-5.58)	3.15 (1.49-6.68)	26 (17.45)	16 (10.53)	2.27 (1.14-4.51)	2.22 (1.10-4.48)	
***P trend***				*p* = 0.0245				*p* = 0.0042	
***Women***									0.0260
Normal	549 (74.29)	587 (72.56)	1.00 (ref)	1.00 (ref)	33 (66.00)	42 (80.77)	1.00 (ref)	1.00 (ref)	
Overweight	137 (18.54)	205 (25.34)	0.72 (0.56-0.91)	0.70 (0.54-0.91)	12 (24.00)	7 (13.46)	2.18 (0.77-6.16)	2.66 (0.83-8.51)	
Obesity	53 (7.17)	17 (2.10)	3.33 (1.91-5.83)	3.51 (1.98-6.24)	5 (10.00)	3 (5.77)	2.12 (0.47-9.53)	2.22 (0.46-10.68)	
***P trend***				*p* = 0.2025				*p* = 0.1047	

^a^cOR, crude odds ratio; CI, confidence interval.

^b^aOR, adjusted odds ratio; CI, confidence interval.

^b^Adjusted for age, educational levels, family allergic diseases history and alcohol consumption.

^c^P value for effect modification by smoking status on the association of BMI category and atopic sensitization in men and women, respectively.

Table 4 presents the association estimates of BMI category with atopic sensitization according to gender and smoking status in those without existing allergic diseases. Among current smoking men, BMI category was significantly related with atopic sensitization (*p*_trend_ *=* 0.0032). Among never smoking men, obesity was positively associated with atopic sensitization (OR, 3.29; 95% CI, 1.37-7.95). In never smoking women, obesity was significantly associated with an increased atopic risk (OR, 3.00; 95% CI, 1.56-5.76). The effect modification by smoking status on the association of BMI category and atopic sensitization was significant in men (*p*_*effect modification*_ *=* 0.0081) but not so in women (*p*_*effect modification*_ *=* 0.0901).

**Table 4:** **ORs and 95% CIs for atopy in relation to BMI stratified by sex and smoking status in subject without allergic diseases**

		Never smokers			Current smokers				Effect
	Case	Control	cOR (95% CI) ^a^	aOR (95% CI) ^b^	Case	Control	cOR (95% CI) ^a^	aOR (95% CI) ^b^	Modification
	N (%)	N (%)			N (%)	N (%)			P value ^c^
***Men***									0.0081
Normal	50 (47.62)	118 (50.86)	1.00 (ref)	1.00 (ref)	47 (47.47)	106 (69.74)	1.00 (ref)	1.00 (ref)	
Overweight	41 (39.05)	102 (43.97)	0.95 (0.58-1.55)	0.98 (0.58-1.65)	35 (35.35)	30 (19.74)	2.63 (1.45-4.78)	2.50 (1.34-4.65)	
Obesity	14 (13.33)	12 (5.17)	2.75 (1.19-6.37)	3.29 (1.37-7.95)	17 (17.17)	16 (10.53)	2.40 (1.12-5.15)	2.44 (1.10-5.38)	
***P trend***				*p* = 0.0671				*p* = 0.0032	
***Women***									0.0910
Normal	313 (74.35)	587 (72.56)	1.00 (ref)	1.00 (ref)	21 (67.74)	42 (80.77)	1.00 (ref)	1.00 (ref)	
Overweight	82 (19.48)	205 (25.34)	0.75 (0.56-1.00)	0.71 (0.52-0.96)	7 (22.58)	7 (13.46)	2.00 (0.62-6.45)	2.35 (0.62-8.95)	
Obesity	26 (6.17)	17 (2.10)	2.87 (1.53-5.37)	3.00 (1.56-5.76)	3 (9.68)	3 (5.77)	2.00 (0.37-10.77)	3.60 (0.47-27.58)	
***P trend***				*p* = 0.5483				*p* = 0.1026	

^a^cOR, crude odds ratio; CI, confidence interval.

^b^aOR, adjusted odds ratio; CI, confidence interval.

^b^Adjusted for age, educational levels, family allergic diseases history and alcohol consumption.

^c^P value for effect modification by smoking status on the association of BMI category and atopic sensitization in men and women without allergic diseases, respectively.

For those subjects who were only allergic to aeroallergens, the results of effect modification of smoking status on the association between obesity and allergic sensitization were similar with those of all subjects allergic to all allergens and subjects without allergic diseases (data not shown). In all subjects and those without allergic diseases, the effect of BMI category on atopic sensitization risk was still stronger in never smokers.

## Discussion

In this hospital-based, case–control study, we demonstrated that not only obesity was associated with atopic sensitization in Chinese males and females, but also such an association was modified by smoking status. We found that the association between obesity and atopic sensitization was stronger in never smokers than in current smokers.

The findings on the association between obesity and atopy in adults in the literature are inconsistent. An epidemiological study of adults in Canada and the US has revealed a significant relationship between obesity and atopy in both women and men [8]. In another birth cohort study, Hancox et al. reported that a raised BMI is associated with allergic sensitization in women but not in men [9]. In contrast, some studies reported contradictory findings [13–17]. Those findings of other populations may not apply to Chinese because allergens, life style and other related factors (such as smoking rate and diet pattern) vary among different regions and cultures [40]. Those variations might have contributed to the inconsistent findings in different populations. In this study, our findings support a positive association between obesity and atopic sensitization in Chinese adults. Additionally, our findings also support a possible sex difference in the relationship between obesity and atopic sensitization which is consistent with a previous study [9]. In our study, OR for obesity was greater in women than in men, but even in men obesity increased risk of allergic sensitization was more than 2 folds. However, our findings are inconsistent with those of another study conducted in China [10], in which no association between BMI category and allergic sensitization was found but percent body fat was found to be associated with a higher rate of allergic sensitization in men than women. The different findings might be due to the differences in the age of study subjects, the panel of allergens and the original objectives between our study and theirs.

Although there are some limitations with the hospital case–control study design, our finding on the relationship between obesity and atopic sensitization being modified by smoking status is intriguing. It suggests the possibility that the association between obesity and atopic sensitization is influenced by smoking status. We demonstrated that the effects of obesity were modified by smoking status in both genders. However, obese adults had more than doubled the odds of having atopic sensitization compared to those with normal BMI regardless of their gender and smoking status. Such associations were not substantially altered in the case of excluding subjects with previously existing allergic diseases. It is not clear why smoking alters the association between obesity and atopic sensitization. There are several possible interpretations. First, current smokers are likely to have lower BMI than non-smokers due to the increased metabolic rate [29–31] or restricting caloric absorption [32], which might reduce the effect of obesity on atopic sensitization risk. Second, a common component of cigarette smoke, such as nicotine, have been shown to suppress various parameters of the immune system [41, 42]. The decreased risk of atopic sensitization in smokers might result partially from the suppressive effects of the components of tobacco on the immune system. Additionally, cigarette smoking could influence atopic sensitization by an indirect mechanism. For example, current smokers might be more likely to have some respiratory tract symptoms, such as chronic cough. It is possible that pathogenic bacteria could alter immune function and affect the incidence of allergic sensitization. Further studies are required to explore the true mechanisms underlying the interaction between obesity and smoking on atopic sensitization. Considering the harmful effect of cigarette smoke, smoking is not encouraged in preventing atopic disorders.

More interestingly, we found that overweight is associated with an increased risk of atopy in current smoking men. Our results should be interpreted with caution. It is worth noticing that BMI is not a perfect measure of excess body fat [43], even though we excluded subjects for whom BMI was not ideal to capture adiposity levels. The power of BMI to distinguish between muscle and excess fat might be limited, which could lead to misclassification of overweight and mild obesity [44]. Moreover, smoking is associated with a decrease in body weight, which might contribute to the observed relationship between overweight and atopy in current smoking men and women.

The strengths of our study include a relatively large sample size of men and women cases and controls, which enable us to look at the effect modification by sex and smoking status on the association of obesity with atopic sensitization. Moreover, obesity was defined according to the current Chinese standard, which could better capture the link between BMI and atopic sensitization in Chinese population. In addition, we were able to assess that the association between obesity and atopic sensitization according to smoking status for the first time.

Our study also had several limitations. First, we did not measure the waist circumference (WC) or the waist-to-hip ratio (WHR). Those measurements are considered to have better correlated with tobacco smoking than BMI [45]. Nevertheless, BMI has been traditionally used as a surrogate measure of adiposity and is the most frequently used diagnostic tool for obesity. Therefore, our findings largely reflect the associations between obesity and allergic sensitization in Chinese population. Second, sample sizes for subgroup analyses for men and women could decrease the power to detect associations. Although we have a relatively large sample size, some subgroup had small numbers of subjects and effect estimates should be interpreted with caution as reflected in the wide confidence intervals. Third, as an observational case–control study, we could not establish a causal link between smoking on the association between obesity and atopy. In addition, selection bias in this study might exist. Atopic patients who came to the allergy department in this hospital were more likely to have a severe symptom of atopy. Finally, we did not obtain data on some important potential confounders, such as physical activity, an important factor for body weight. Since our study was conducted in a single hospital in Harbin, China, the generalizability of our findings to Chinese in other regions or to other populations remains to be further assessed.

## Conclusions

Obesity is significantly associated with the risk of atopic sensitization in male and female adults in China. In addition, smoking status modified the effect of BMI on atopy risk. Although the association between BMI category and atopic sensitization is less strong in current smokers than in non-smokers, obesity increases the risk of atopic sensitization for obesity by more than two folds regardless of their gender and smoking status. Our findings have important public implications for developing strategies of preventing allergic sensitization.

## Abbreviations

BMI: Body mass index
%BF: Percent body fat
IgE: Immunoglobulin E
SIgE: Specific immunoglobulin E
OR: Odds ratio
cOR: Crude odds ratio
aOR: Adjusted odds ratio
CI: Confidence Intervals
WC: Waist circumference
WHR: Waist-to-hip ratio.
